# Predictive Analysis of Diabetes-Risk with Class Imbalance

**DOI:** 10.1155/2022/3078025

**Published:** 2022-10-11

**Authors:** Ahmed I. ElSeddawy, Faten Khalid Karim, Aisha Mohamed Hussein, Doaa Sami Khafaga

**Affiliations:** ^1^Information Systems Department, Arab Academy for Science and Technology -AASTMT, Cairo, Egypt; ^2^Department of Computer Sciences, College of Computer and Information Sciences, Princess Nourah bint Abdulrahman University, Riyadh 11671, Saudi Arabia; ^3^Information Systems Department, Faculty of Computers and Artificial Intelligence Helwan University, Cairo, Egypt

## Abstract

Diabetes type 2 (T2DM) is a common chronic disease, increasingly leading to many complications and affecting vital organs. Hyperglycemia is the main characteristic caused by insufficient insulin secretion and poses a serious risk to human health. The objective is to construct a type-2 diabetes prediction model with high classification accuracy. Advanced machine learning and predictive model techniques are utilized to achieve cutting-edge techniques for the early diagnosis of diabetes. This paper proposes an efficient performance model to predict and classify the minority class of type-2 diabetes. The impact of oversampling and undersampling approaches to reduce the effect of an unbalanced clas*s* has been compared to classification performance algorithms. Synthetic Minority Oversampling (SMOTE) and Tomek-links techniques are applied and examined. The outcomes were then compared to the original unbalanced dataset using an artificial neural network (ANN) predictive model. The model is compared with other state-of-the-art classifiers such as support vector machine (SVM), random forest (RF), and decision tree (DT). The tuned model had the best accuracy of 92.2%. The experimental findings clearly manifest the improvement in accuracy and evaluation metrics in terms of AUC and F1-measure using the SMOTE oversampling strategy rather than the baseline and undersampling schemes. The study recommends adopting dynamic hyperparameter optimization to further improve accuracy.

## 1. Introduction

Diabetes mellitus is described as a fatal disease since it is a well-recognized lifelong illness. It causes the body to generate less insulin and raises blood sugar levels, leading to disruptions in the regular functioning of organs including the eyes, nerves, kidneys, and heart.

The global prevalence of diabetes among individuals over 18 years of age increased from 4.7 percent in 1981 to 8.5 percent in 2014 [1], with the number of persons with diabetes growing from 108 million in 1981 to 422 million in 2014. Diabetes is predicted to affect 642 million people (1 in 10) by 2040, with 46.5% of those undiagnosed [2]. According to scientists, diabetes is influenced by both hereditary and environmental variables. Early identification and treatment can help to reduce disease-related complications and risk factors.

As the healthcare business produces and creates a large amount of useable data, such as patient data, electronic medical records, and diagnostic and treatment data, this may serve as a valuable resource for knowledge discovery [3, 4] to aid decision-making and reduce costs.

In order to determine if a certain parameter is at risk of getting diabetes given the independent variables, several studies were conducted but shown to be inaccurate. For instance, insulin and BMI have not been linked to a history of type-2 diabetes. However, obesity is not necessarily related to a higher BMI [5]. It is necessary to integrate different observations for early diagnosis. However, when several factors were combined for diabetes prediction, the techniques used could not produce good results.

Medical data, on the contrary, is recorded over a long period of time and therefore frequently has unbalanced data sets. Unbalanced data is considered an unequal distribution of samples among the various classes. The unbalanced nature of medical data makes it difficult to mine various resources. Although significant progress has been made in machine learning, creating efficient algorithms that depend on unbalanced data remains a difficult challenge [6]. Therefore, the main purpose of this study was to find and include the best data preprocessing approaches before using the processed data for the machine learning model's training.

The proposed solutions to overcome the class imbalance problem fall into two categories: [a] data-level solutions, which modify data distribution and yield an improved set with balanced data distribution; and [b] algorithmic level, which modify and optimise the accuracy of the classifier [7, 8]. Sampling is the basic data-level solution and can be either undersampling (removing the majority of class instances) or oversampling (increasing the number of minority classes). Oversampling can result in overfitting and increase complexity and execution time. Undersampling may discard many potentially relevant data at random that raises the risk of losing critical data [8, 9]. Undersampling may be helpful in massive data applications to reduce computational time. Resampling methods have also been used to address the imbalance problem in conjunction with class overlap, noise occurrence, and/or borderline examples in the data set.

Most resampling methods rely on the *k* nearest neighbor (KNN) rule [7, 10], either by eliminating instances of two classes that are far from the decision boundary to reduce duplication as in condensing or by removing those that are close to the boundary for generalization as in filtering [11]. Similarly, Tomek-links are used to eliminate instances from the majority class since, if two examples form a Tomek link, then either one of them is noise or both are borderline.

To address these problems, the current study handles class imbalance and overlap by employing SMOTE oversampling and Tomek-links undersampling to obtain a subset from the majority of instances and avoid eliminating instances that may help to develop knowledge.

Considering the importance of early detection of T2DM, machine learning and statistical principles are used to generate predictive power, allowing it to automatically extract knowledge from massive databases as well as identify valuable patterns and interrelations [6]. The presented paper introduces ANN, SVM, RF, and decision tree classifiers for diabetes onset prediction. MLP is a form of ANN that can learn from experience and extract key features from inputs that contain extra, unnecessary data. The performance of a neural network is affected by too many hidden layers, resulting in an overfitting issue [12]. SVM is a statistically supervised ML classifier for binary classification problems that uses a sequence of mathematical functions called kernels to transform the input into the proper format. With huge datasets, it might be difficult to choose the proper kernel function, and it takes a long time to train. A decision tree is a supervised machine learning approach that does not need extensive data preprocessing. However, there are a few exceptions. There are certain constraints. It is not stable; a tiny change in the data can have a big impact on the final estimates, and complexity appears with huge datasets. As a result, the preparation and analysis take a long time [12]. Considering these factors, the proposed framework is suggested to produce more accurate results in comparison to other literature.

The contributions of the paper are as follows:

1. Preprocessing techniques were applied that included filling in missing values, outliers' treatment, feature selection, data transformation, and handling imbalanced data for homogeneity.
2. This study demonstrated a combination application of machine learning, SMOTE oversampling, and Tomek-links undersampling techniques for the treatment of class imbalances, followed by the application of normalization to the data.
3. Implementations and trials were performed on the ANN model using a grid search technique to ensure optimal selection of hyper-parameters optimization with minimal time execution and on the SVM model to select the best kernel with optimal parameters.
4. The performance of ANN classification of different resampling datasets was compared to achieve the appropriate balancing technique that yields the most accurate results.
5. Three classifiers, namely, SVM, RF, and decision tree (c-Tree), were introduced and compared to ensure the high quality of the model's performance. Furthermore, a comparison analysis was conducted with other approaches.

The rest of the paper is organized as follows: Section 2 includes a review of related work. The method is proposed in Section 3 with data collection and parameter setup.  Section 4 describes the experimental results. The study's findings are discussed and concluded in Section 5.

## 2. Related Work

Early and onset detection is a critical step in the prevention and control of diabetes. Using a Pima Indian Diabetes dataset from the UCI repository, advanced machine learning prediction algorithms have been suggested in the literature.

Gupta et al. [13] utilized a feature selection strategy and k-fold cross-validation to increase the prediction performance of diabetes. The SVM classifier achieves higher accuracy when compared to the naive Bayes model. A comparative study of diabetes classification was conducted by Choubey et al. [14] on PIMA India and a local diabetes dataset. PCA and LDA were used for feature selection. They applied AdaBoost, KNN regression, and the radial basis function and revealed that when combined with classification methods, both may assist in increasing accuracy and eliminating undesired variables. On the PIMA dataset, Ahuja et al. [15] did a comparison evaluation of multiple techniques. They found that MLP outperformed NB and DT in terms of accuracy. Mohapatra et al. [16] employed MLP to identify diabetes and reached a 77.5 percent accuracy without presenting comparisons.

A stacking-ensemble technique was suggested by Singh and Singh [17]. They trained four base modules using the bootstrap technique and cross-validation, including SVM, decision tree, RBF, and poly SVM, but with no feature selection and comparison. On PIMA and breast cancer datasets, Kumari et al. [18] constructed a diabetes prediction system that uses a stack of random forest, logistic regression, and naive Bayes to compare their outcomes, and their system yields 79 percent. Khandegar and Pawar [19] employed PCA to choose attributed features, followed by a neural network (NN) classifier, with 92.2% accuracy. Zhu et al. [2] used K-means to cluster the results after applying PCA, and LR was used to classify them, yielding an accuracy of 89.0%. Moreover, SVM, J48, KNN, and random forest (RF) classifiers were compared by Kandhasamy and Balamurali [20]. The accuracy rate was 73.82% for J48 and reached 100% for KNN and RF. Mercaldo et al. [21] used two algorithms, Greedy Stepwise and BestFirst, to find the discriminating features that improve classification performance. Six algorithms are used. The Hoeffding Tree approach yielded the greatest accuracy of 75.5%, with a recall of 76.2%. Mohebbi et al. [22] employed an MLP neural network and CNN with an LR activation function. The diabetic dataset is comprised of continuous glucose monitoring signals that yield 77.5% accuracy using the CNN classifier. Ramesh et al. [23] used the Recurrent Neural Network (RNN) to predict Type 1 and Type 2 diabetes. The dataset utilized was the Pima Indian dataset and the predicted accuracy for diabetes type 1 was 78% while it was 81% for type 2. Lekha [24] used modified CNN to predict individuals' breath signals, composed of five diabetic patients of type 1, nine diabetic patients of type 2 and 11 healthy patients. The performance was evaluated using area under curve and was 0.96.

While the class imbalance solution poses a significant limitation, undersampling causes the removal of important data and oversampling causes overfitting. Moving on toward an undersampling strategy: Mustafa et al. [9] have proposed a hybrid method of MultiBoost ensemble and random undersampling to solve the class imbalance problem. Kubat and Matwin [25] proposed OSS (one-sided selection) that provides an undersample of the majority class instances that are redundant (border instances). Barrela et al. [26] defined a new cluster-based OSS technique (ClusterOSS) to overcome the limitations of OSS. The majority of class instances are selected and clustered by k-means. Then, OSS is applied to the instances closest to the center of every cluster. Borderline and noisy cases are removed using Tomek-links. Mani and Zhang [27] proposed a scheme to use the KNN classification method to select the instances to be eliminated during undersampling. Undersampling is combined with clustering to propose a cluster-based undersampling technique [28]. The idea of Tomek-links is to uncover border cases, whereas Hart [29] defined the condensing CNN undersampling technique to detect redundant cases. The minority class is grouped into K clusters by the K-means algorithm in the Fast_CBUS technique developed by Ofek et al. [30], and for each cluster, a comparable number of examples from the majority class that are near the minority class instances are selected. Raghuwanshi and Shukla [31] used an extreme learning machine (ELM) undersampling classifier to create ensemble subsets of the majority class that yielded 80.5% accuracy. Roy et al. [32], combine both SMOTE-Tomek to balance the Pima diabetes dataset using ANN and had achieved accuracy of 98%. Guzmán-Ponce et al. [11] proposed two undersampling strategies that combine DBSCAN clustering to eliminate noisy samples and refine the decision boundary with a minimal spanning tree (MSA) algorithm to deal with the class imbalance.

Moving on to oversampling strategies, Han et al. [33] proposed a borderline SMOTE strategy for producing synthetic examples from borderline cases with significant misclassification costs. Barua et al. [7] clustered the synthetic data generated after applying MWMOTE-SMOTE. To overcome the problem of class imbalance, Gustavo et al. [34] developed a mix of undersampling and oversampling. Ensemble learning gives a more precise solution to the problem of class imbalance. AdaBoost [35] used ensemble methods to apply different weights to both successfully classified and misclassified minority samples. To properly classify minority class instances, Chawla et al. [36] presented a mix of sampling and ensemble learning. Wu and Chang [37] used the SVM to create a class-boundary alignment approach. Stefanowski and Wilk [38] reported that the identification of minority classes is only influenced by class imbalance when associated with additional data challenges such as outliers and redundant data. Therefore, outliers must be taken into consideration when handling unbalanced data Table 1.

## 3. Methodology

In this section, we introduce the diabetes dataset as a binary classification problem to differentiate whether a patient is suffering from the disease or not. This approach includes multiple preprocessing steps for cleaning data, feature extraction, and algorithms to predict the onset of diabetes.

### 3.1. Datasets

The Pima Indians' diabetes dataset was obtained from the public UCI data repository [45]. All were female. Of the 768 total numbers, 268 (35%) were diabetes instances and 500 (65%) were nondiabetic instances. It includes eight independent variables; the first attribute was the number of times they have had pregnancies. The second was the plasma glucose concentration in a 2 h oral glucose tolerance test (mean value of 141 suffered from the disease), followed by the diastolic blood pressure (mm·Hg), fourth was triceps skin fold thickness (mm), then 2 h serum insulin (u U/ml), followed by body mass index (weight in kg/(height in *m* ^ 2)) with a mean value of 35.14 suffering from the disease and 30 not suffering, seventh was diabetes pedigree function, and finally was age (years). The dependent variable (class) is defined as (1, 0) for the presence or absence of diabetes. In order to analyze the impact of the attributes on the occurrence of diabetes, Table 2 shows the positive association between the attributes and the class. Using the t-test, glucose, BMI, pregnancy, and age had a significant effect (p value 0.05). While the etiologic reasons for NIDDM in Pima Indians are likely to be comparable to those in other ethnic groups, the genes that predict predisposition to the illness are more frequent in Pimas, according to a study of 200 normal, nondiabetic Pima Indians dataset [46].

#### 3.1.1. Feature Selection Using Relative Odds

A logistic regression model is used to characterize the risk factors for developing T2DM. The odds ratio generated provides a ranking of the explanatory variables to help determine the output [47]. Diabetes is higher in the age group (<25 years) in comparison to the older group (>40 years) with an odds ratio = 6.5, as shown in Table 3. Women with one to three pregnancies are at high risk of developing diabetes (odds ratio = 1.6). Normal-weight women had a nearly 8-fold increased risk of diabetes, while women with low blood pressure are three times more likely to become diabetic. Data showed that abnormal insulin secretion is a major factor, and women with normal 2-hour glucose concentration have a 7-fold elevated risk of developing diabetes. These findings were in accordance with [46], who reported that insulin resistance is a main risk factor for noninsulin-dependent diabetes mellitus development. The incidence of diabetes was higher in normal BMI women than in overweight subjects that may be due to a genetic predisposition factor.

### 3.2. Proposed Framework

**Figure 1:**
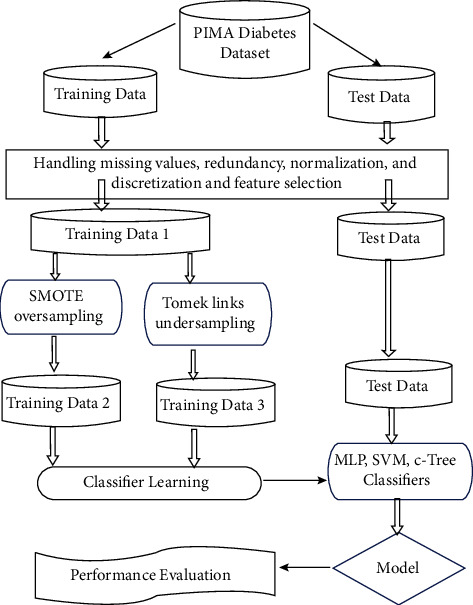
Framework for the proposed methodology.

We used the software R Programming Version 3.4 for data analysis and machine learning. Initially, the median value is used to handle missing values in the dataset, followed by handling each attribute's outliers. Then, ranking of the top risk variables for developing diabetes was performed using the random forest model (accuracy of 94%) and the Boruta package (accuracy of 78.6%). The essential features (glucose, BMI, insulin, age, and skin fold thickness) are in line with existing standards and have a significant influence on developing diabetes [19, 39]. The data was then split into a proportion of 80% training and 20% testing. The training set was balanced by SMOTE oversampling and Tomek-links undersampling. Then, each training set was normalized by scaling and embedded into an ANN model where the tuning process and hyperparameters were chosen. ANN was compared to other classifiers, SVM, and tree models, as shown in Figure 1. Performance metrics are carried out by accuracy, sensitivity, specificity, ROC curves, F1-score, and Kappa.

### 3.3. Preprocessing of Data

Noise in the dataset causes inconsistency, which leads to inaccurate outputs. Data cleaning, handling of outliers, and data balancing are preprocessing stages that are discussed here.

#### 3.3.1. Data Cleaning

Some attributes have zero values that include glucose, BMI, insulin, skin thickness, and blood pressure. The missing values were replaced by the median value in reference to the outcome parameter in a process called imputation with outlier corrections. For example, missing values for glucose levels with median values of 110 were assigned to outcome “0” and median values of 140 to outcome “1”.

#### 3.3.2. Dimensionality Reduction

Feature selection is used to reduce the number of attributes while still yielding the same number of attributes. The quality of data is measured by the good correlation of features with the target class and the independent correlation to each other. random forest and the Boruta library in *R* were used as wrapper algorithms for the selection of variables. Glucose, BMI, and insulin are the most important features.

#### 3.3.3. Handling Outliers

A data preparation method known as the interquartile range (IQR) is used to find outliers and extreme values. By splitting a rank-ordered dataset into four (Q1, Q2, Q3, Q4) equal portions, or “quartiles,” it calculates dispersion. Whereas Q2 is the median, the IQR is the middle half of the data that lies between the upper Q3 and lower quartiles of Q1.

(1) $$\begin{array}{c} IQR=Q3–Q1\text{,}\\ Q3+1.5*IQR<Outliers<below\;Q1+1.5*IQR\text{.} \end{array}$$

We replace the extreme values with median values since the median is more robust than the mean and is the middle rank irrespective of its value. Moreover, we consider the upper and lower boundaries for the outlier's replacement.

#### 3.3.4. Normalization of Data

A min–max normalization is used to scale all numeric parameters to the range [0, 1]. Therefore, before training, apply (equation 1) to avoid the effect of variables with a broader range of values.

(2) $$\begin{array}{c} \text{Normalized}\left(Y\text{new}\right)=\frac{\left(Y-Y_{\min}\right)}{\left(Y_{\max}-Y_{\min}\right)}\text{.} \end{array}$$

#### 3.3.5. Stabilization of Data

**Figure 2:**
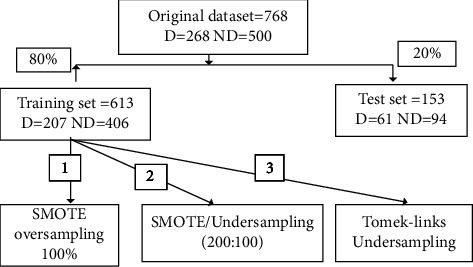
Generation of training and testing datasets. (*SMOTE: Synthetic minority oversampling technique).*

The imbalanced distribution of classes was biased toward the negative class (majority class), leading to the misclassification of the positive class (minority class) as shown in Figure 2. The following techniques are used to handle this problem:

*(1) Tomek-Link Technique*. The Tomek-link undersampling method is a refinement of the Condensed Nearest Neighbor (CNN) [48] aimed at reducing boundary occurrences that have a tendency to be misclassified. If there is no sample xk such that d (xi, xk) < d (xi, xj), two samples xi and xj with class (xi) = class (xj) are shown to produce a Tomek-link pair. In other words, instances that form a Tomek-link pair generate noise in the data distribution. Outlier and duplicate instances, in addition to boundary instances, all contribute to the problem of class imbalance. An outlier is a case that goes beyond the decision boundary, possibly increasing the misclassification error. Redundant instances are those that have the same information as one another. The Tomek-links technique uses the collective elimination of outlier, boundary, and redundant instances from the majority class to ensure informed deletion while also reducing loss of information. The goal is to clarify the border between the minority and majority classes so the minority regions become more distinct.

*(2) The Synthetic Minority Oversampling Technique (SMOTE)*. In SMOTE methods, the number of minority class instances was increased, and thus there is no loss of information from the original dataset. The distance is determined by the Euclidean distance. New samples are created (Ynew), then the distance is multiplied by a number between 0 and 1 (*σ*) [49].

(3) $$\begin{array}{c} Ynew=Yi+\left(Yj\;–\;Yi\right)*\;\sigma \text{.} \end{array}$$

The SMOTE finds the *k* nearest neighbors of a given minority data instance from the neighbourhood by utilizing the k-NN method. The length of the line segment connecting two locations xi and xj equals the Euclidean distance between them. Each new instance is created by multiplying the differences (diff) between the relevant characteristics of the chosen neighbour instance and the original instance by a random value (gap) between 0 and 1 and adding them to the features (Di) of the original minority instance (Algorithm 1). This helps define the derived instance's end position [49], which might be the same as the original minority instance, a randomly picked neighbour, or anywhere in between.

*(3) Combination of SMOTE and Undersampling*. By randomly removing samples from the majority class, the majority class is undersampled until it reaches the proportion of the minority class. According to [32], a combination of SMOTE and undersampling or oversampling yields better results than SMOTE alone. The adjusted dataset will contain twice as many entries from the minority class if the majority class is undersampled by 200 percent. Therefore, by combining both oversampling and undersampling, the training dataset would have the minority class “smoted” and the majority class “undersampled”, as shown in Table 4.

### 3.4. Machine Learning Algorithms

#### 3.4.1. Artificial Neural Network (ANN)

Interconnected neurons with numeric weights that carry messages between each other are referred to as ANNs. Updated weights of the learning method and activation function that convert weighted inputs to the learning method's output. In the R-neural net package, standard neural networks' “backpropagation” with three layers was utilized [50], with the input, hidden, and output layers, respectively, and repetition of 5. The Resilient Backpropagation algorithm of type rprop^+^ was used as the training set [51]. The rprop is utilized by multilayer perceptrons (MLP) to minimize errors by applying a learning rate to the weights in the reverse direction of the gradient. The network is then assessed on a set of test variables after the training set. (4) used back propagation MLP to adjust weight update using gradient descent [52] and learning rate *α*.

(4) $$\begin{array}{c} \Delta wijr=-\;\frac{\alpha r\delta y}{\delta x}\text{.} \end{array}$$

#### 3.4.2. Support Vector Machines (SVMs)

**Figure 3:**
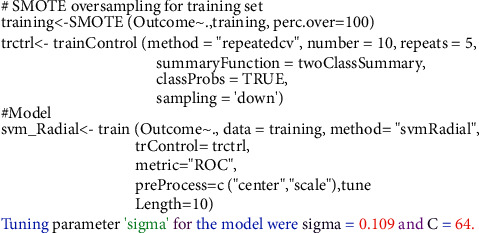
SVM model for SMOTE oversampling training dataset using R software.

**Figure 4:**
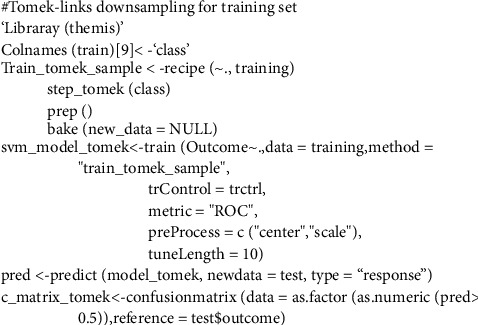
SVM model for Tomek-link undersampling training and test datasets.

The SVM algorithm splits the data into two groups by performing an n-dimensional hyperplane. The SVM algorithm splits the data into two groups by performing an n-dimensional hyperplane, a kernel function with a sigmoid shape. A two-layer perceptron neural network is quite similar to the SVM model. The kernels are a set of training approaches for polynomial, RBF, and MLP classifiers in which the network's weights are computed by solving a quadratic programming problem with linear criteria [53], rather than nonconvex, as in traditional “neural network” training. The goal of SVM is to partition datasets into classes so that the largest marginal hyperplane may be found [54] because the biggest margin yields the best test case. In this paper, we construct our model using the radial basis function nonlinear kernel (RBF). Various kernel types were used in the SVM network training. By applying the “e1071” package in R Figures 3 and 4, the best performance was attained for a network using an RBF kernel. It is utilized to create completely nonlinear hyperplanes.

(5) $$\begin{array}{c} k\left(x,x'\right)=\exp\;\left(\frac{-\;\left\|x-x^{'}\right\|}{2\sigma^{2}}\right)\text{,} \end{array}$$

where *x* & x' are feature space vectors. *σ* is an unbounded parameter. The critical choice is the choice of the parameters.

*(1) Hyperparameter Optimization*. Tunning of cost and epsilon parameters were selected as (ranges of the list (epsilon = seq (0, 1, 0, 1), and cost = 2 ^ (2 : 9)).

If *n* = number of training examples, *m* = number of features, and *k* = number of support vectors, the computational complexity of training SVM using Big O notation is O (*n* ^ 2) and for testing is O (*k∗m*) [55].

**Figure 5:**
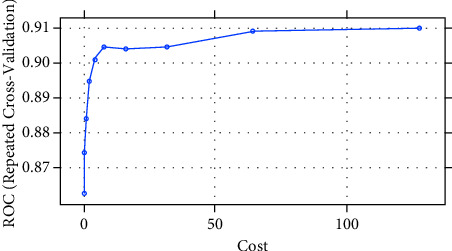
AUC for SVM-RBF after training SMOTE 100%, sigma = 0.1210985 and C = 128.

**Figure 6:**
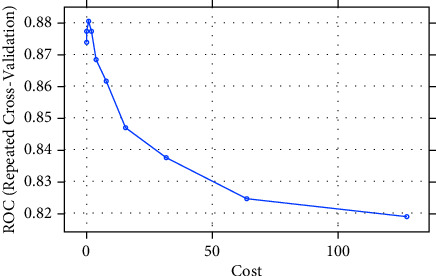
AUC for SVM-RBF after training Tomek-links, sigma = 0.127 and C = 1.0.

Table 5 shows a comparison between different SVM kernels. Tunning parameters (sigma, cost) and ROC were used to select the optimal model using 10-fold cross-validation. The best accuracy was obtained after oversampling using SVM with the RBF kernel. The largest AUC value was obtained after training with a 100% SMOTED of 0.96 and an accuracy of 0.974, as shown in Figure 5. On the other hand, on applying Tomek-links on the training set (Figure 6), the accuracy of SVM was 0.88 with an AUC of 0.89.

#### 3.4.3. Random Forest (RF)

Random forest constructs numerous independent decision trees and aggregates them (Algorithm 2), typically yielding a more accurate and precise outcome. The final prediction output by RF is the category that received the most votes across the forest. Similar hyper-parameters to those of a decision tree or bagging classifier are present. The reality that underlies RF simplicity is the overlap of random trees. RF produces more accurate results on big datasets, and more random trees may be produced by establishing a random threshold for all features rather than locating the most precise threshold. The overfitting problem is also resolved by this approach [56].

#### 3.4.4. Decision Tree

For regression analysis, recursive binary splitting is a prominent method. Exhaustive search algorithms frequently employed to generate such models have two major drawbacks: overfitting and bias selection towards covariates with multiple splits or incomplete data. Although pruning can be improved with overfitting, the bias of feature selection still has a significant impact on the use of structured tree regression models. Conditional inference trees (CTree) are nonparametric regression trees found in the *R* package. The association between outcomes and covariates is investigated by the CTree to make unbiased covariate selections at various levels. CTree differs from the CART and C4.5 algorithms [58] in that it does an exhaustive search over all possible splits before picking the covariate with the best split.

**Figure 7:**
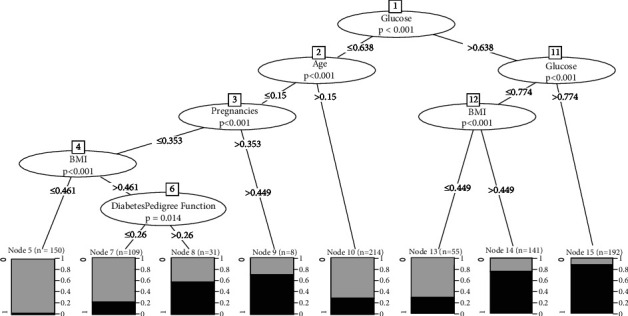
Conditional inference tree for training set after oversampling. The Bonferroni significant *p* values are presented for each inner node, and the proportion of result is displayed for every terminal node. *Diabetes* *=* *1, NonDiabetes* *=* *0*.

The tree model shown in Figure 7 shows the following:

1. Root: glucose (most significant feature).
2. Glucose ≤ 0.638, age ≤ 0.15, pregnancy ≤ 0.353, BMI > 0.461, anddiabetes pedigree function ≤0.26 (*n* = 109, err = 20.2%).
3. Glucose > 0.638 and glucose > 0.774 (*n* = 192, err = 87.3%).

### 3.5. Assessment Measures for Class Imbalance

Accuracy as an evaluation metric can be misleading in imbalanced data. The G-mean is an average obtained from both minority and majority classes, the higher its value, the better, as shown in Table 6. Other metrics include the F-measure, which provides good classifier performance in the minority class. The balance between sensitivity and specificity using area under the curve-receiver operation curve (AUC-ROC) of one is a perfect model [59].

## 4. Experimental Results and Discussion

The experimental findings were evaluated and analyzed using the metrics in Table 6.

### 4.1. Analysis and Evaluation of ANN

To avoid overfitting or underfitting problems, we first perform grid search to select the best parameters for training the model.

#### 4.1.1. ANN Hyperparameter Optimization

Grid search was applied to datasets (Table 7) before and after resampling in this study to choose the optimal parameters and improve and lower the training error. Setting the tuning grid with 10-fold cross-validation The tuning parameters are weight, the number of hidden layers, and hidden units; decay is the weight decay; and there are three tuning values (0, 0.01, and 0.1); the learning rate is set to 0.01. Size is the number of hidden units per layer. The number of layers maintains a balance between high bias and variance and has been selected to be two layers. The batch size is 32, and the number of iterations is 250 (Table 8). The next optimizer is gradient descent to find local minima, manage the variance, and adjust the model's parameters [52].

**Figure 8:**
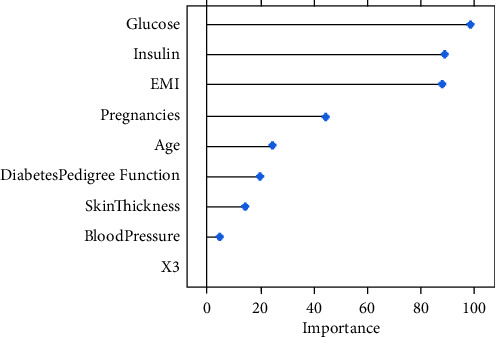
Features selection priority using the varImp function on ANN evaluation.

Initially, the diabetes dataset without oversampling is classified using (ANN). Then the data after resampling is experimented with ANN. The best performance was achieved with four layers (8, c (5, 2), 1) and 10^−2^ learning rate. The most significant features used in the generation of the model were sorted using the varImp function in the NeuralSens package in *R* (Figure 8). The run-time execution was shown in (Tables 7 and 9).

The proposed models have been developed and tested on a PC having the following specifications: Microsoft Windows 10 operating system, i5-core processor @ 2.40 GHz, and 6 GB of RAM.

### 4.2. Computational Complexity

The time complexity is determined by using Big O notation. For ANN trained with gradient descent (backpropagation) runs for n iterations, with n layers each with n neurons, delta error, weights as well as adding feedforward propagation is O (*n*^e) [52].

In our model, the backdrop contains 4 layers (1 input, 2 hidden, and 1 output) denoted i, j, k, and l with delta weight update in *t*-training time and n epochs is O (nt *∗* (ij + jk + kl)). This is the same as the feed-forward pass network. The result is O (nt *∗* (ij + jk + kl)).

**Figure 9:**
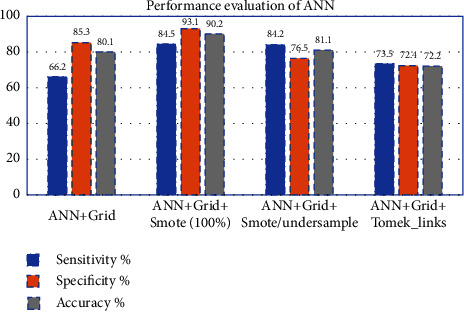
Performance evaluation of ANN classifier before and after resampling.

**Figure 10:**
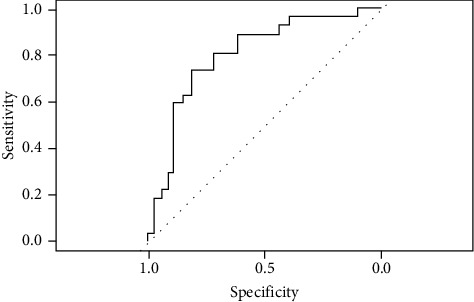
AUC of 0.89 ANN classifier after SMOTE 100% oversampling.

The performance of the ANN classifier after overoptimized hyper-parameters was shown in Table 9, using different training sets. The accuracy after training ANN with (SMOTE 100%) was 90.2%, sensitivity was 84.5%, and specificity was 93.1% (Figure 9) that exceeds other data sets. The training time was 290 sec and the AUC was 0.89, as shown in Figure 10.

Finding the optimal algorithm with ideal hyper-parameters in ANN is a challenge, as it requires too much computing time. The author [60] reported that the Antlion optimizer outperforms grid search in choosing the optimal hyper-parameters in the stroke dataset using DNN within a limited amount of time.

**Figure 11:**
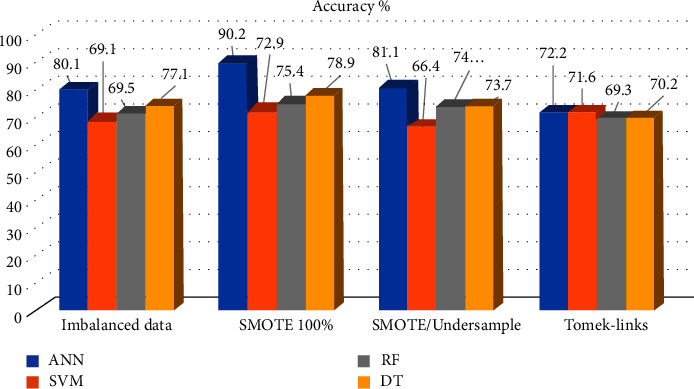
Comparison performance of all classifiers using accuracy%.

The dataset with and without resampling are then experimented with other machine learning algorithms such as SVM, RF, and DT. The percent of improvement in performance after applying to resample was considered in Table 10 and Figure 11. The ANN model shows an improvement in accuracy after applying SMOTE 100%oversampling. On the other hand, the Tomek-link technique yields 72.2% lower than the original training set, which was 80.1%, as shown in Figure 9.

**Figure 12:**
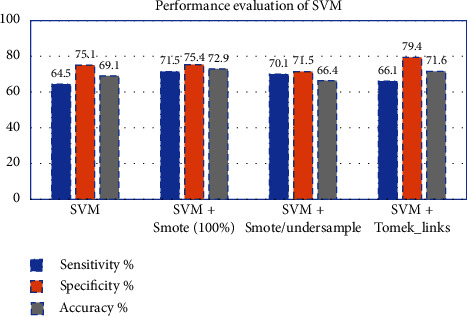
Performance evaluation of SVM classifier before and after resampling.

Hyperparameter optimization s improve SVM after SMOTE 100%oversampling, the accuracy was 72.9%, and AUC of 0.73. By applying Tomek-link undersampling, the accuracy was improved to 71.6%, except for the F1-measure, which shows no significant change in Figure 12.

**Figure 13:**
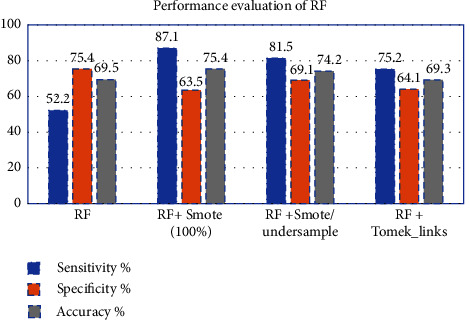
Performance evaluation of RF classifier before and after resampling.

By using the training set without resampling, RF shows 52.2% sensitivity, 75.4% specificity, and an accuracy of 62%. While after resampling, the accuracy was improved and achieved 75% in simulated (100%) and only 65% in Tomek-links, as shown in Figure 13.

**Figure 14:**
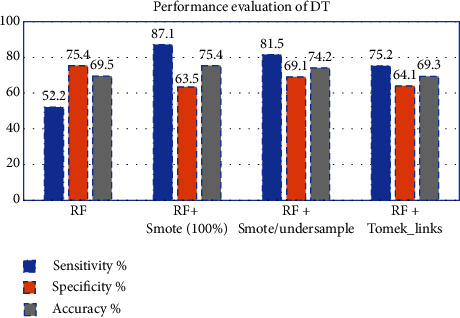
Performance evaluation of DT classifier before and after resampling.

In this study, the CTree model is influenced by blood glucose levels, BMI, and pregnancies. Insulin and BMI, on the other hand, appear to have a greater impact on diabetics than the other factors as shown in Figure 7. The accuracy after training SMOTE 100% oversampling was 87.8%, SMOTE/undersample was 83.4%, and after applying Tomek-links was 75.5%, while test results were shown in Figure 14.

The overall summary of the best training set was shown in Table 11. The ?ndings revealed that the proposed ANN outperforms traditional models with respect to precision, recall, F1-score, and accuracy of the model.

### 4.3. Area under the Receiver Operating Characteristics (AUC-ROC) Curve

ROC is a probability curve, and AUC represents the degree or measure of separability. We considered AUC as part of the performance evaluation. The greater the area, the better the model with FP rate = 0 plotted against TP rate = 1. In contrast, G-mean represents the performance in absolute values.

Figure 10 shows the AUC of optimized oversampled ANN at 0.89, which means that the model can discriminate between positive and negative classes by 89%. ANN was the best classifier, exceeding other classifiers with AUC.

The optimization of the hyper-parameters (cost and sigma) of SVM classifiers using the RBF kernel was also evaluated by AUC-ROC. Figure 5 shows the AUC of SVM after training SMOTE (oversampled 100%) of 0.905, while after the test it was 0.73, as in Table 10. Figure 6 shows the AUC of SVM after undersampling with Tomek-links that achieved 0.88, while the test set was 0.7 in Table 10.

**Figure 15:**
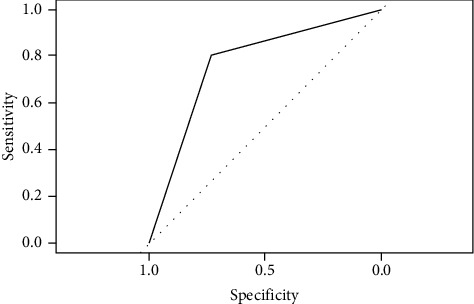
AUC of 0.78 CTree classifier after SMOTE 100% oversampling.

Figure 15 shows the AUC of CTree, SMOTE oversampling also exceeds the value of 0.78.

Our work is compared to that of Devi et al. (2017). They implemented abnormal Tomek links to undersample the majority and overlapping regions in the diabetes dataset. They applied the optimization ratio in both Feed Forward NN and SVM (Cost = 10, Sigma = 0.1). The accuracy of Tomek-links for ANN was 76.0%, while combined SMOTE-Tomek gave 75.0% with an AUC of 0.66. While the accuracy of Tomek-links for SVM was 85% and combined SMOTE-Tomek gives 75.0% only with an AUC of 0.72.

### 4.4. Comparison of the Effectiveness of the ANN model to Benchmark Research for Predicting Patients with Diabetes

To evaluate the effectiveness of the ANN model, the performance is compared with other literature using the same diabetes dataset. ANN accuracy performs better than other studies, as shown in Table 12.

## 5. Conclusions

The current approaches involve inaccurate classification techniques as they do not consider several crucial data preparation steps that can significantly improve the level of performance. Several risk assessments for diabetes early detection are reported in the current study. The relationship between the attributes has been analyzed using conventional methods, and the inferences were predicted using analytical approaches. Using machine learning involves preprocessing steps of filling in missing data and class imbalances that might lead to misclassification. The training data were balanced using SMOTE oversampling, Tomek-links undersampling, and a combination of SMOTE/undersample modeled with ANN. To characterize the risk of developing type-2 diabetes, the odds ratio, the Boruta filter, and the varImp function were applied to rank the important variables. Our results were consistent with the guidelines and previous studies [32], where insulin resistance and elevated BMI were shown to be the major risk factors. Oversampling was performed on the training set using a SMOTE ratio of 100%. The prediction performance was higher in all models, with AUC and F1-measures ranging between 0.69 and 0.89. Furthermore, the results of Tomek-links were not considerably better than the original training set in all classifiers. A grid search method is used to track the maximum values of the parameters in order to optimise the hyper-parameters using ANN. The cost-sensitive method was applied to SVM for optimization. The ANN and SVM's sensitivity and accuracy were greatly enhanced by the oversampling stage. The ANN and SVM's sensitivity and accuracy were greatly enhanced by the oversampling stage. The predictive performance of the CTree classifier is unaffected by rebalancing. The AUC of 0.89 and accuracy of 90.2% indicate that ANN is the best model in both the oversampled and test datasets. The SMOTE oversampling increases the learning capability and improves performance rather than the Tomek-links technique.

To conclude, model-based oversampling can be utilized to identify individuals at high risk of getting diabetes and provide a timely response in treatment to women in our community aged 21 and older. Our limitation in this study is the lower number of samples. To lower the risk and effects of type 2 diabetes, the research proposed that more regulated attributes and frequent follow-up be offered, particularly during pregnancy. Future studies can be performed using sensitivity analysis and regularizations to select the most significant features based on deep learning for the early prediction of diabetes diseases. In addition, hyper-parameters can be optimized dynamically.

## Figures and Tables

**Algorithm 1:**
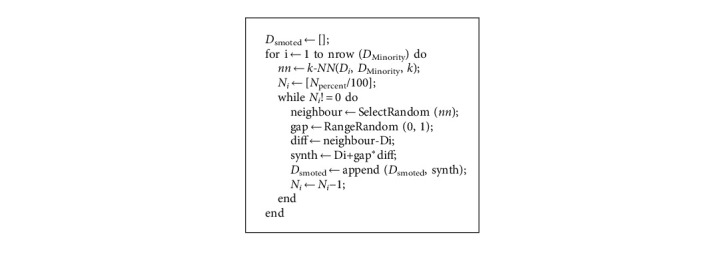
SMOTE [10].

**Algorithm 2:**
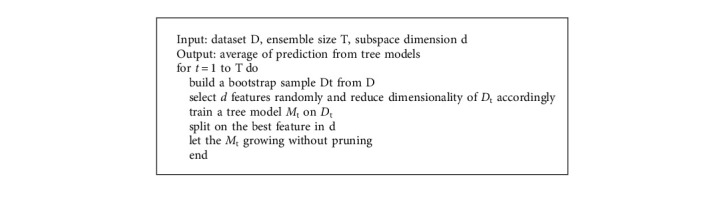
Random Forest Pseudocode [57].

**Table 1:** Summary of related works.

Reference	Approach	Algorithm	Significance and limitations
Zhu et al. [2]	Dimensionality reduction of Pima diabetes dataset	PCA, K-means, LR	Accuracy: 97.40%

Devi et al. [8]	Class imbalance and class overlap on Pima dataset. Eliminate missing values.	FFNN outperforms NB, SVM	Accuracy: 82.0%

Gupta et al. [13]	Dimensionality reduction of Pima diabetes dataset	K-fold CV SVM outperforms NB	Accuracy: 81.1, 79.2%, no comparable studies

Choubey et al. [14]	Dimensionality reduction of Pima diabetes dataset by PCA + LDA	AdaBoost, classification via regression (CVR), RBF, KNN	PCA-CVR: 91% accuracy with excessive feature selection.

Singh and Singh [17]	Data preprocessing of Pima diabetes dataset	Ensemble model (NSGA-II) outperforms SVM, DT, RBF and poly-SVM	Accuracy: 83.8%.
			Reduction techniques and comparability not applied

Kumari et al. [18]	Data preprocessing of Pima diabetes dataset	Stacking model of RF, NB, LR	Accuracy of 79.04%.
			Need more effective preprocessing steps

Khandegar and Pawar [19]	Dimensionality reduction of Pima diabetes dataset	PCA + NN	Accuracy: 92.2%

Kandhasamy and balamurali [20]	Data preprocessing of Pima diabetes dataset	RF outperforms (J48 DT, KNN, SVM)	Accuracy: 100%.
			No comparability with other studies

Mercaldo et al. [21]	Dimensionality reduction of Pima diabetes dataset	HoeffdingTree outperforms J48, MLP, JRip, BayesNet, RF	Accuracy: 75.5%

Mohebbi et al. [22]	Classification methods with grid search	CNN outperforms MLP	Accuracy: 77.5% no comparison with other studies

Roy et al. [32]	Prediction of diabetes: (i) Missing values by median (ii) Combined SMOTE-Tomek techniques.	(i) Lrgbm (ii) ANN	Accuracy 91.0% 98.0%

Jhaldiyal and Mishra [39]	Dimensionality reduction of Pima diabetes dataset	PCA + SVM outperforms PCA + REP	Accuracy: 93.66%
			No comparability with other studies

Maniruzzaman et al. [40]	Comparative approach in Pima dataset	GPC outperforms LDA, QDA, NB	Accuracy: 81.97%

Butt et al. [41]	Classification and prediction on Pima dataset	MLP outperforms RF, LR	Accuracy: 86.08%
		LSTM outperforms MA, LR	Accuracy: 87.26%
			No feature selection

Nnamoko and Korkontzelos [42]	SMOTE oversampling of outliers in Pima diabetes dataset	NB, SVM, ripper, **C4.5**	77.0, 77.7, 83.6**, 89.5%**

Zeng et al. [43]	Handle class imbalance in Pima dataset	K^*∗*^, SVM, BN, AdaBoost, C4.5, RBF network, LR, LMT	**80.33,** 77.45, 78.23, 77.56, 77.45, 75.58, 78.23, 79.11%

Wang et al. [44]	ADASYN oversampling	NB-ADASYN-**RF**	87.10%

**Table 2:** Feature characteristics of diabetes in Pima Indians' dataset.

Feature name	Mean ± SD	Diabetes (*N* = 268)	Nondiabetes (*N* = 500)	*P* value
Age (years)	33.24 ± 11.8			
(<25)		31 (11.8%)	188 (38.0%)	<0.05
(25–30)		53 (20.0%)	124 (25.2%)	
(30–35)		42 (15.5%)	50 (9.6%)	
(35–40)		34 (12.9%)	39 (8.3%)	
(>40)		108 (40.0%)	99 (19.9%)	

No. of pregnancies	3.8 ± 3.4			
Never		38 (14.0%)	73 (14.0%)	<0.05
(1–3)		75 (28.2%)	238 (45.6%)	
(4–6)		60 (22.0%)	115 (25.7%)	
(>6)		95 (35.8%)	74 (14.7%)	

Insulin level (u U/ml)	79.8 ± 15.2			
(Less than 200)		221 (83.0%)	458 (92.0%)	<0.05
(More than 200)		47 (18.0%)	42 (8.0%)	

BMI (kg/m^2)	31.9 ± 7.9			
Normal		7 (3.0%)	108 (22.0%)	<0.05
Overweight		122 (46.0%)	239 (46.9%)	
Obesity		139 (52.0%)	153 (31.1%)	

Blood pressure (mm·Hg)	69 ± 19.4			
Low (<65)		46 (18.0%)	155 (31.4%)	<0.05
Normal (65–85)		173 (64.6%)	288 (57.2%)	
High (>85)		49 (17.4%)	57 (11.4%)	

Glucose (mg/dL)	120.9 ± 32			
Normal (<140)		131 (49.9%)	438 (88.6%)	<0.05
High (>140)		137 (50.1%)	62 (11.4%)	

Skin fold (mm)	20.5 ± 16			
(<20)		103 (38.6%)	235 (47.4%)	<0.05
(20–40)		123 (45.9%)	217 (43.0%)	
(>40)		42 (15.5%)	48 (9.6%)	

Pedigree function	0.47 ± 0.3			
(<0.5)		163 (60.8%)	319 (63.8%)	<0.05
(0.5–1.0)		87 (32.5%)	145 (29.0%)	
(>1.0)		18 (6.7%)	36 (7.2%)	

**Table 3:** Multiple logistic regression model in diabetes mellitus dataset.

Independent variables	Dependent variable (diabetes)				
	B	Odds ratio	Lower limit	Upper limit	Significant risk
Age (years)					
(<25)	−1.2	6.55	4.1	10.6	0.0
(25–30)	−0.7	2.55	1.6	3.9	0.0
(30–35)	−0.9	1.29	0.78	2.1	0.0
(35–40)	−1.15	1.26	0.72	2.13	0.0
(>40)	1	1	Ref		

Number of pregnancies					
Never	1	1	Ref		
(1–3)	0.69	1.66	1.0	2.6	0.0
(4–6)	0.46	1.0	0.6	1.6	0.02
(>6)	0.92	0.4	0.2	0.6	0.0

Insulin level (u U/ml)					
(Less than 200)	0.9	1	Ref		
(More than 200)	1.5	2.5	1.5	3.7	0.0

BMI (kg/m ^ 2)					
Normal	−2.5	8.01	3.6	16.9	0.0
Overweight	1	1	Ref		
Obesity	0.1	0.6	0.39	0.69	0.3

Blood pressure (mm·Hg)					
Low (<65)	−1.3	2.9	1.7	4.8	0.0
Normal (65–85)	−1.2	1.4	0.9	2.2	0.0
High (>85)	1	1	Ref		

Glucose (mg/dL)					
Normal (<140)	−0.4	7.3	5.0	11.0	0.0
High (>140)	1	1	Ref		

Skin fold (mm)					
(<20)	−0.1	1.3	0.9	1.8	0.1
(20–40)	1	1	Ref		
(>40)	−1.0	0.6	0.4	1.0	0.0

**Table 4:** Characteristics of the datasets after preprocessing steps.

Dataset	Positive	Negative	Total
Baseline	268	500	768
Smote (100%)	414	414	828
SMOTE/undersample (200 : 100)	648	432	1080
Tomek-links	216	329	545

**Table 5:** Comparative analysis after applying different SVM kernels on training sets.

Type	Kernel type	Training imbalanced data	SMOTED oversampling (100%)	SMOTE/Undersample (200 : 100)%	Tomek-links
Accuracy of SVM model	Linear	69.4%	73.0%	80.4%	80.2%
	Linear grid	82.6%	90.1%	85.7%	84.6%
	Radial basis Function (RBF)	76.0%	97.4%	96.6%	88.0%
		*C* *=* *0.5, S* *=* *0.1*	***C*** ***=*** ***64, S*** ***=*** ***0.109***	*C* *=* *64, S* *=* *0.118*	*C* *=* *1.0, S* *=* *0.12*

Hyperparameter (Cost = “c,” sigma = “s”) for SVM-RBF kernel.

**Table 6:** Performance metrics for the classification model.

Performance metric	Formula
Precision	TP/(tp + FP)
Recall (sensitivity)	TP/(tp + FN)
Specificity (true negative rate)	TN/(TN + FP)
F1-score	2 *∗* (precision *∗* Recall)/(Precision + recall)
Accuracy	(TP + TN)/(TP + TN + FP + FN)
G-mean	$\sqrt{Sensitivity\;*\;Specificity}$

TP = true positive, TN = true negative, FP = false positive, FN = false negative.

**Table 7:** Evaluation of best performance of ANN using grid search.

No. of neurons	No. of iterations	Decay	Accuracy	AUC	Time
50	250	0.01	0.752	0.801	2.45 min
30	250	0.01	0.725	0.786	2.30 min
10	250	0.01	0.861	0.90	2.10 min
**9**	**250**	**0.01**	**0.940**	**0.927**	**1.30 min**
3	250	0.01	0.79	0.801	50 sec

**Table 8:** Optimized parameters used for training ANN.

No.	Parameters	Values
1	Loss function	Binary cross entropy
2	Optimizer	Gradient descent (backpropagation)
3	Algorithm	“rprop+”
4	Activation function	“Logistic”
6	Stepmax	1e + 06
7	Learning rate	0.01
8	Metrics	Accuracy

**Table 9:** The ANN model's accuracy, error rate, and training time.

Training data	Test accuracy (%)	MSE	Training time (sec)
Original set	0.801	0.06	31.83
Smote (100%)	0.902	0.02	310
Smote/undersample	0.811	0.03	**290**
Tomek-links	0.722	0.08	228.6

MSE: mean square error (loss function).

**Table 10:** Comparison between multilayer perceptron, support vector machine, RF, and decision tree based on various metrics in test data set.

Model	Sensitivity	Specificity	F1-score	Kappa	Precision	Accuracy	ROC	G-mean
ANN + Gridsearch	0.662	0.853	0.682	0.51	0.704	0.801	0.71	0.75
ANN + Gridsearch + Smote (100%)	0.845	0.931	0.871	0.66	0.903	0.902	0.89	0.89
ANN + Gridsearch + Smote/undersample	0.842	0.765	0.774	0.52	0.721	0.811	0.73	0.80
ANN + Gridsearch + Tomek_links	0.735	0.724	0.643	0.42	0.575	0.722	0.64	0.73

SVM	0.645	0.751	0.609	0.38	0.657	0.691	0.69	0.70
SVM + smote (100%)	0.715	0.754	0.675	0.29	0.707	0.729	0.73	0.73
SVM + Smote/undersample	0.701	0.715	0.582	0.29	0.606	0.664	0.69	0.71
SVM + Tomek_links	0.661	0.794	0.593	0.44	0.675	0.716	0.70	0.72

RF	0.522	0.754	0.652	0.33	0.652	0.695	0.62	0.63
RF + smote (100%)	0.871	0.635	0.787	0.40	0.713	0.754	0.75	0.74
RF + smote/undersample	0.815	0.691	0.694	0.38	0.614	0.742	0.62	0.75
RF + Tomek_links	0.752	0.641	0.543	0.38	0.494	0.693	0.65	0.7

DT	0.711	0.801	0.691	0.22	0.674	0.771	0.74	0.75
DT + smote (100%)	0.789	0.764	0.669	0.41	0.627	0.789	0.78	0.78
DT + Smote/undersample	0.812	0.682	0.674	0.31	0.584	0.737	0.70	0.74
DT + Tomek_links	0.762	0.664	0.784	0.39	0.793	0.702	0.74	0.71

**Table 11:** Summary of evaluation metrics using SMOTE (100%).

	Classifier	Sensitivity	Specificity	F1-score	Kappa	Precision	Accuracy	ROC	G-mean
Test dataset smote 100%	ANN	0.84	0.93	0.87	0.66	0.903	0.902	0.89	0.89
	SVM	0.71	0.75	0.67	0.19	0.70	0.729	0.73	0.73
	RF	0.87	0.63	0.78	0.40	0.71	0.754	0.75	0.74
	DT	0.78	0.76	0.66	0.36	0.627	0.789	0.78	0.78

**Table 12:** Comparative results of ANN with previous studies based on accuracy%.

Author	Approach	Algorithm	Accuracy%
Alam et al. [5]	Prediction of diabetes	ANN	75.7%
	Median values and NB imputation		
Pradhan et al. [61]	Prediction of diabetes with classifier comparisons	ANN	85.09
Guldogan et al. [62]	Prediction of diabetes:	MLP	78.1%
	Missing values deleted	RBF	76.8%
Ahuja et al. [15]	Prediction of diabetes:	MLP	78.7%
	Missing values by median		
Ramezani et al. [63]	Predicting diabetes with reducing number of features from 8 to 5	LANFIS	88.05%
Our work (ANN)	Predicting diabetes with preprocessing steps + SMOTE 100%	ANN	90.2%

## Data Availability

Datasets analyzed during the current study are available from the UCI repository, 452 datasets were derived from the following public domain resources: [https://www.kaggle.com/da- 453 tasets/uciml/pima-indians-diabetes-database].
